# Association of comorbidities and medications with risk of asthma exacerbation in pediatric patients: a retrospective study using Japanese claims data

**DOI:** 10.1038/s41598-022-08789-7

**Published:** 2022-04-01

**Authors:** Shotaro Maeda, Shigetoshi Kobayashi, Kenzo Takahashi, Satoshi Miyata

**Affiliations:** 1 Teikyo University Graduate School of Public Health, 2-11-1 Kaga Itabashi-ku, Tokyo, 173-8605 Japan; 2 Medical Affairs, Kyorin Pharmaceutical, Tokyo, Japan; 3 Department of Pediatrics, Teikyo University School of Medicine, Tokyo, Japan

**Keywords:** Asthma, Paediatric research

## Abstract

Asthma exacerbation impairs the quality of life of pediatric patients and negatively impacts future respiratory function and health economics. Several risk factors associated with exacerbations have been identified; however, most studies report the risk of each factor. Therefore, this study aimed to evaluate the risk of each factor and a combination of factors. We performed a retrospective cohort study using Japanese claims data and extracted factors associated with exacerbations using multivariate Cox proportional hazards regression and stepwise method. Risk scores were then calculated from the extracted factors and validated by tenfold cross validation. Of the 1,748,111 asthma patients in the database, the data of 14,980 were extracted, and 1988 (13.3%) had exacerbation. Factors associated with asthma exacerbation were age of 3–5 years, exacerbation history before cohort entry date, allergic rhinitis, chronic sinusitis, otitis externa, blepharitis, upper respiratory infections, urticaria, LTRA prescription, were determined. A four-level risk score was calculated from 9-factors and the AUC derived from cross validation was 0.700. Most factors extracted in our study are consistent with those of previous studies. We showed that combining each factor is more helpful in assessing the increased risk of asthma exacerbation than assessing each factor alone.

## Introduction

In 2015, a national school survey using the International Study of Asthma and Allergies in Childhood (ISAAC) questionnaire showed that the prevalence of asthma was 10.1% in 6–8 year old children and 8.2% in 13–15 year old children^1^. In a worldwide survey using the same ISAAC questionnaire, the prevalence of asthma was 13.7% and 11.6% in 6–7 year old and 13–14 year old children, respectively, indicating that asthma is a common disease in children^2^.

In the treatment and management of asthma, exacerbation is a critical challenge. When exacerbations occur due to insufficient management, they not only pose health problems, such as impaired quality of life and affecting patient prognosis, including future deterioration of respiratory function and increased mortality^3,4^, but also place a burden on medical economics, since the medical costs related to exacerbations account for a large proportion of the direct costs of asthma treatment^5^.

To prevent exacerbations, the essential means required are avoidance and elimination of the risk factors in daily life, appropriate assessment of the clinical features of exacerbation-prone patients, and the continuous control of inflammation and airway dilation with appropriate pharmacotherapy. There are several reports on the factors associated with exacerbations, including respiratory infection and concomitant diseases, such as allergic rhinitis and chronic sinusitis, as well as the living environment, such as tobacco, dust mites, and pollen, as well as the severity and control of asthma^6–8^. However, it has also been reported that the occurrence of exacerbations cannot be sufficiently predicted by the severity and control status alone^9^. Although respiratory function tests using spirometry and fractional exhaled nitric oxide (FeNO) are useful in assessing the control status and predicting exacerbations, it is difficult to perform these tests on younger children because of the difficulty in handling them^10^. In addition, the reimbursement system in the Japanese health insurance system makes it difficult to perform FeNO frequently in children. Therefore, there is a need for useful predictors composed of information that can be easily obtained during usual practice. The results of a large stakeholder analysis of childhood asthma reported by Mathioudakis et al. in 2020 showed that the highest priority unaddressed clinical questions related to asthma treatment and management are biomarkers related to the diagnosis, prognosis, and treatment, which are important not only for clinicians, researchers, and pharmaceutical companies involved in asthma treatment, but also for the affected children and their families^11^.

Research on the elucidation of the clinical features of asthma exacerbations in children is also called "Therapeutic Orphan" due to the difficulties in actively conducting large-scale prospective studies due to the challenges in research design and dosage setting, such as the high hurdle of obtaining consent and the diversity of their backgrounds^12^.

In contrast, as a research method other than actual clinical practice, several studies have evaluated the exacerbation factors and the usefulness of treatment in children with asthma by the secondary use of routinely collected data, such as administrative health insurance claims data^13–15^. However, asthma exacerbations may not be caused by specific factors alone, but rather by the combination of various backgrounds and environments of the affected children, which may determine the individual exacerbation risk. However, to date, there have been no reports of such studies using claims data.

Therefore, this study aimed to identify the factors associated with exacerbations of greater than moderate severity in pediatric asthma that are highly generalizable and easily obtained during routine clinical practice through a comprehensive analysis of the population included in a large claims data set in Japan. In addition, we evaluated the composite risk, which is increased by the accumulation of individual risk factors, by calculating the risk score.

## Methods

### Study design and, data source

This study was a retrospective cohort study that used Japanese claims data. The database used in this study was MediScope®, which consists of medical, dental, dispensing, and diagnostic procedure combination (DPC) data collected from approximately 70 health insurance associations in Japan, and includes information such as age, sex, diagnosis, medication, medical treatment, and the region of members and their families^16^. The database contains approximately 7.2 million unique patients, representing 5.4% of the Japanese population. In this study, we used a dataset of asthma patients (N = 1,748,111) who had J45 (asthma) or J46 (asthma attack severity) in the International Classification of Diseases 10th revision (ICD-10) between May 2014 and April 2019. Since the data used in this study are already anonymized claims data, it is impossible to confirm the authenticity of the listed disease names; however, the validity of the diagnosis was subjectively reviewed by one of the authors, KT, a pediatrician.

### Study subjects

In the current study, the patients who met all of the following criteria were included in the analysis.

1. Patients who had at least one asthma diagnosis and prescription of asthma medication between the period from May 2016 and June 2018. The first day on which the above aforementioned diagnosis and treatment occurred in the same month during this period is defined as the “cohort entry date (CED)”.

(2) Aged at 5 years or younger at the cohort entry date.

(3) Patients with at least two diagnoses of asthma and prescription on the same claim during the period from the day after the cohort entry date to one year after the cohort entry date (the “follow-up period”). In addition, to ensure that children with suspected respiratory infections were not included in the cohort entry date for asthma, antibiotics (ICD-10 code: J01) and asthma treatment were not prescribed on the same day (following eligibility criteria as well).

(4) Patients who had at least two asthma diagnoses and prescriptions on the same claim during the period from the day before the cohort entry date to one 1 year before the cohort entry date (the “look back period”).

(5) Patients for whom data existed after the follow-up period and before the look-back period.

**Figure 1:**
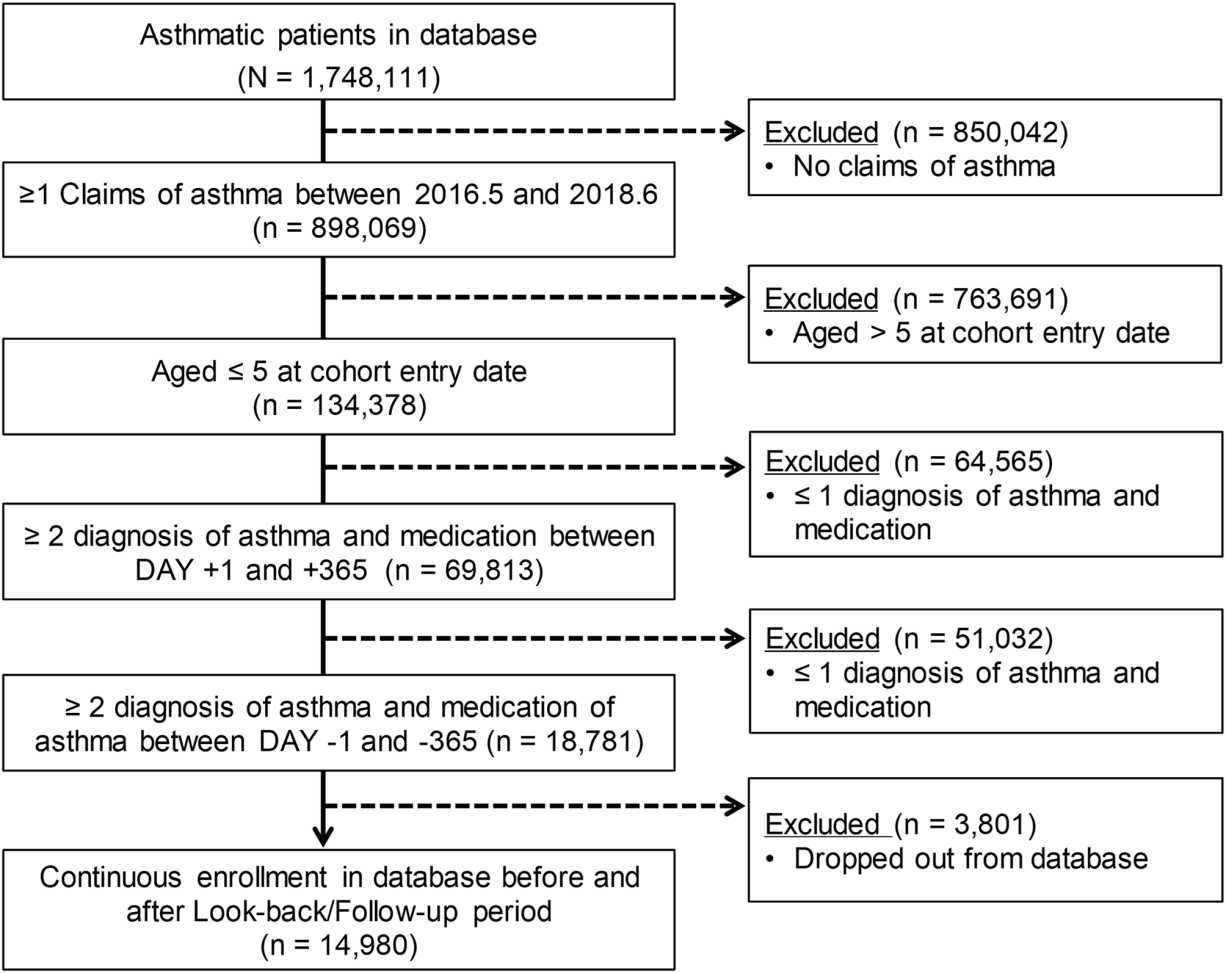
Flow of eligible subjects. CED, cohort entry date.

Regarding the third criterion, in actual practice in Japan, asthma is sometimes diagnosed in non-asthmatic patients to prescribe asthma medication that has no indication for the common cold or allergic rhinitis in children. Therefore, to ensure the validity of the fact that the subjects of the present analysis were “asthmatic” patients from the standpoint of clinical relevance, we set the criterion of having received the diagnosis and treatment of asthma at least twice each in the year before and after the date of cohort participation. The analysis subjects who met all the eligibility criteria are shown in Fig. 1.

### Study endpoints

The primary endpoint of this study was to evaluate the association between exacerbation events that occurred during the follow-up period and each subject’s background of the subjects using a Cox proportional hazards analysis. In addition to the risk assessment of the individual factors, the comprehensive risk of exacerbation, which is increased by the combination of multiple factors, was assessed by calculating a risk score.

### Outcomes

The outcomes were defined using ICD-10 codes for diagnostic information, anatomical therapeutic chemical classification system codes (ATC codes) for prescriptions, and Japan-specific standardized procedures^17^. Since only the claims data were available in this study, we were not able to use information on various biomarkers for “exacerbation” or “assessment of severity of exacerbation” as indicated in the International consensus on (ICON) pediatric asthma guidelines and other guidelines^18^. Therefore, we defined medical interventions that can be assumed to be closely associated with exacerbations as surrogate indicators of exacerbations. Asthmatic exacerbations were defined as hospitalization and administration of intravenous corticosteroids, occurring at the same time as the claims of a diagnosis of asthma. The number of days from the cohort entry date to the onset of exacerbation was calculated.

### Variables

We extracted information on age, sex, and region at the cohort entry date, as well as information on diseases and asthma medications that occurred in the record during the look-back period. Since exacerbations during the look-back period were considered to be closely related to exacerbations during the follow-up period, history of exacerbations before the date of cohort entry was considered as a variable.

The odds ratios (ORs) for the occurrence of exacerbations of each code were calculated for all ICD-10 codes (2298 codes in the subdivision) that occurred during the look-back period of the eligible subjects. We extracted codes with an OR of 95% CI lower limit greater than 1 or an upper limit of less than 1. Subsequently, we excluded codes that were used for the reimbursement of prescriptions or clinical tests (e.g., K769 liver disease, K590 constipation) and codes that were considered to be associated with some events and not appropriate as background factors (e.g., E86 decreased fluid volume). Finally, the codes were aggregated based on clinical judgment (Supplementary Table S1).

For treatment, the prescription of DSCG (disodium cromoglycate), ICS (inhaled corticosteroid), LTRA (leukotriene receptor antagonist), and SABA (short acting beta-2 agonist) records in the 3 months prior to the cohort entry date were tabulated. Of these, “with prescription” was defined as when a record of prescription had occurred for 28 days or more. As for the prescription of DSCG, since DSCG is often used as a bulking agent for nebulized SABA inhalation, a combined variable with SABA was included in the analysis. Age, sex, exacerbation-free period in the look-back period, extracted and aggregated history and complications, and treatment items were used as the explanatory variables in subsequent statistical analyses.

### Statistical analyses

To show the distribution of each patient background in the groups with and without exacerbations, the mean (SD) or median (IQR) were calculated for continuous variables, and the presence/absence and percentage (%) were calculated for categorical variables as summary statistics. Chi-squared tests and standardized differences between groups were performed for each aggregated background^19^. Crude ORs and 95% CIs were calculated using the presence of exacerbations as the dependent variable and the occurrence of each ICD-10 code as the independent variable to identify the patients’ background factors for risk assessment.

In the primary analysis, a Cox proportional hazards analysis was performed to evaluate the association between exacerbations and the patient background, using the period (number of days) between the cohort entry date and the exacerbation. Sensitivity analysis was performed using classification and regression trees (CART) to classify patient background from simply “with” or “without” to “more than” or “less than” the number of diagnoses and number of exacerbation-free days before CED. CART is a type of binary decision tree that constructs a homogeneous subpopulation by the recursive partitioning of data with a set of branching conditions for covariates^20^. For the survival tree model, *p*-value adjusted log-rank statistic was applied for the evaluation. The purpose of using CART to binarize medical history and other variables in the regression analysis is that, in Japan, the names of diseases listed in claims data are often not the same as the names of diseases given for some prescriptions or tests. The factors that could not be bifurcated by CART were leveled by their presence or absence (0 and > 1).

In the secondary analysis, a risk score was calculated to assess the combined risk of multiple patient backgrounds. First, we conducted a univariate Cox proportional hazards analysis with time to exacerbation (days) as the dependent variable to identify the background factors included in the risk score. The extracted variables were then discarded using the stepwise method (both forward and backward selection method based on BIC); additionally, and the hazard ratios (HRs) obtained for the selected variables were rounded to the first decimal place, and the integer values were used as the scores. Variables for which the lower limit of the 95% confidence interval of the hazard ratio was greater than 1 were assigned a positive score, and variables for which the upper limit was less than 1 were assigned a negative score. Because the risk score itself ranges widely from − 1 to 14 points, we classified it into levels according to the size of the risk in consideration of clinical usability. The total score for each patient was entered into CART with the time to exacerbation as the dependent variable and leveled by risk level, and a Kaplan–Meier curve was created for each level to show the cumulative risk of exacerbation. Finally, to validate the risk score, we split the analysis subjects into training data (80%) and test data (20%), performed a tenfold cross-validation, and evaluated the validity of the model using Accuracy and area under the receiver operating characteristic curve (AUC).

For all statistical analyses, R version 4.10 was used for all statistical analyses (R Core Team (2021). R: A language and environment for statistical computing. R Foundation for Statistical Computing, Vienna, Austria. URL https://www.R-project.org/). The significance level of the test was set at a two-tailed 5% level; and the confidence interval was calculated as 95%.

### Ethics

This study used only anonymized claims data and was exempted from ethical review under the Japanese ethical guidelines; additionally , and the Teikyo University Medical Research Ethics Committee determined that no referral to the Ethics Review Committee was necessary^21^. This study was conducted in accordance with the Ethical Guidelines for Medical and Health Research Involving Human Subjects (Ministry of Education, Culture, Sports, Science and Technology, and Ministry of Health, Labor and Welfare of Japan)^21^ and reported in accordance with the recommendations of the Strengthening the Reporting of Observational Studies in Epidemiology (STROBE)^22^.

## Results

### Baseline characteristics

The background characteristics of the patients are shown according to the presence or absence of exacerbations during the follow-up period (Table 1). Of the 14,980 patients included in the analyses, 1988 patients (13.3%) had exacerbations. The frequency and proportion of exacerbations by age, exacerbation-history, medical history/complications, and asthma treatment are shown in Table 2.

**Table 1:** Baseline characteristics.

	Non-exacerbation group (n = 12,992)		Exacerbation group (n = 1988)		Std diff	*P*-value*
**Sex, n (%)**						
Female	5471	42.1%	747	37.6%	0.09	< 0.001
Male	7521	57.9%	1241	62.4%		
**Age, n (%), years**						
1	1725	13.3%	240	12.1%	0.04	0.259
2	2542	19.6%	363	18.3%	0.03	
3	2855	22.0%	460	23.1%	0.03	
4	3018	23.2%	481	24.2%	0.02	
5	2852	22.0%	444	22.3%	0.01	
**Area, n (%)**						
Hokkaido	528	4.1%	113	5.7%	0.08	< 0.001
Tohoku	1019	7.8%	170	8.6%	0.03	
Kita-kanto, Koshin	1001	7.7%	136	6.8%	0.03	
Minami-kanto	4574	35.2%	665	33.5%	0.04	
Tokai	811	6.2%	217	10.9%	0.17	
Hokuriku	544	4.2%	64	3.2%	0.05	
Kinki	1761	13.6%	237	11.9%	0.05	
Chugoku	509	3.9%	80	4.0%	0.01	
Shikoku	266	2.1%	70	3.5%	0.09	
Kyushu	1979	15.2%	236	11.9%	0.10	
**Exacerbation type, n (%)**						
Hospitalization						
Yes	0	0.0%	637	32.0%		
No	12,992	100.0%	1351	68.0%		
Systemic corticosteroids						
Yes	0	0.0%	1482	74.6%		
No	12,992	100.0%	506	25.5%		

*Std-diff* standardized difference.

*P-values were derived from chi-square test.

**Table 2:** The frequency and proportion of exacerbations by age, exacerbation-history, medical history/complications, and asthma treatment.

Variables, n (%)	Non-exacerbation group (n = 12,992)		Exacerbation group (n = 1988)		Std diff	*P*-value*
**Age, years**						
Greater than 2	8725	67.2%	1385	69.7%	0.05	0.027
**Exacerbation history**						
Yes	1545	11.9%	1033	52.0%	0.95	< 0.001
**Medical history and complications, n (%)**						
Allergic rhinitis						
Yes	10,099	77.7%	1695	85.3%	0.19	< 0.001
Chronic sinusitis						
Yes	3360	25.9%	669	33.7%	0.17	< 0.001
Otitis media						
Yes	5438	41.9%	960	48.3%	0.13	< 0.001
Otitis externa						
Yes	5304	40.8%	1001	50.4%	0.19	< 0.001
Blepharitis						
Yes	3605	27.8%	672	33.8%	0.13	< 0.001
Upper respiratory infections						
Yes	9752	75.1%	1599	80.4%	0.13	< 0.001
Lower respiratory infections						
Yes	11,437	88.0%	1840	92.6%	0.15	< 0.001
Influenza						
Yes	7110	54.7%	1175	59.1%	0.09	< 0.001
Dermatitis						
Yes	10,684	82.2%	1660	83.5%	0.03	0.168
Urticaria						
Yes	2990	23.0%	512	25.8%	0.06	0.007
Intestinal viral infection						
Yes	696	5.4%	153	7.7%	0.09	< 0.001
Unspecified site viral/bacterial infection						
Yes	1338	10.3%	311	15.6%	0.16	< 0.001
**Asthmatic medication, n (%)**						
ICS						
Yes	1944	15.0%	407	20.5%	0.14	< 0.001
LTRA						
Yes	4921	37.9%	794	39.9%	0.04	0.079
DSCG and SABA						
SABA with/without DSCG	1877	14.5%	445	22.4%	0.21	< 0.001
DSCG without SABA	146	1.1%	25	1.3%	0.01	

*ICS* inhaled corticosteroid, *DSCG* disodium cromoglycate, *LTRA* leukotriene receptor antagonist, *SABA* short acting beta-2 agonist, *Std-diff* standardized difference.

*P-values were derived from chi-square test.

### Risk assessment

Of the 14,980 patients included in the analysis, 1988 patients (13.3%) had exacerbations, and the median number of days between the cohort entry date and the onset of exacerbation was 119 days (IQR, 56, 206). The univariate analysis showed that the factors significantly associated with exacerbation were age (vs. aged 2 years or younger; HR 1.12, 95%CI 1.01–1.23), sex (vs. female; 1.19, 1.09–1.31), exacerbation history during the look-back period (vs. no exacerbation; 6.46, 5.92–7.06, allergic rhinitis (vs. no; 1.61, 1.42–1.82), chronic sinusitis (vs. no; 1.42, 1.29–1.55), otitis media (vs. no; 1.27, 1.17–1.39), otitis externa (vs. no; 1.43, 1.31–1.43), blepharitis (vs. no; 1.31, 1.19–1.43), upper respiratory infections (vs. no; 1.34, 1.20–1.50), lower respiratory infections (vs. no; 1.64, 1.38–1.94), influenza (vs. no; 1.18, 1.08–1.29), urticaria (vs. no; 1.15, 1.04–1.27), intestinal viral infection (vs. no; 1.44, 1.22–1.70), unspecified site viral/bacterial infection (vs. no; 1.57, 1.39–1.77), ICS prescription (vs. No; 1.42, 1.28–1.59), SABA prescription with/without DSCG (vs. no DSCG and SABA; 1.65, 1.48–1.83).

The results of the multivariate Cox regression analysis on the factors selected by the stepwise method showed that the background factors significantly associated with increased exacerbations were age (vs. aged at 2 years or younger; 1.14, 1.03–1.25), exacerbation history during the look-back period (vs. no exacerbation; 6.29, 5.75–6.89), allergic rhinitis (vs. no; 1.35, 1.18–1.53), chronic sinusitis (vs. no; 1.15, 1.04–1.26), otitis externa (vs. no; 1.22, 1.12–1.34), blepharitis (vs. no; 1.12, 1.02–1.23), upper respiratory infections (vs. no; 1.12, 1.00–1.26), Urticaria (vs. no; 1.11, 1.00–1.23), LTRA prescription (vs. No; 0.87, 0.79–0.94). The Harrell’s c-statistic for the multivariate Cox regression model was 0.718 (Table 3).

**Table 3:** Variables selected by stepwise method and risk score.

Variables	Reference	Unadjusted model			Adjusted model			*P*-value*	Score
		HR	LCL	UCL	HR	LCL	UCL		
**Age, years**									
Greater than 2	2 or less	1.12	1.01	1.23	1.14	1.03	1.25	0.009	1
Sex									
Male	Female	1.19	1.09	1.31	1.09	1.00	1.20	0.054	
Days without exacerbation, days									
Yes	No	6.46	5.92	7.06	6.29	5.75	6.89	< 0.001	6
**Medical history and complications**									
Allergic rhinitis									
Yes	No	1.61	1.42	1.82	1.35	1.18	1.53	< 0.001	1
Chronic sinusitis									
Yes	No	1.42	1.29	1.55	1.15	1.04	1.26	0.006	1
Otitis media									
Yes	No	1.27	1.17	1.39					
Otitis externa									
Yes	No	1.43	1.31	1.57	1.22	1.12	1.34	< 0.001	1
Blepharitis									
Yes	No	1.31	1.19	1.43	1.12	1.02	1.23	0.016	1
Upper respiratory infections									
Yes	No	1.34	1.20	1.50	1.12	1.00	1.26	0.045	1
Lower respiratory infections									
Yes	No	1.64	1.38	1.94					
Influenza									
Yes	No	1.18	1.08	1.29					
Dermatitis									
Yes	No	1.08	0.96	1.22					
Urticaria									
Yes	No	1.15	1.04	1.27	1.11	1.00	1.23	0.042	1
Intestinal viral infection									
Yes	No	1.44	1.22	1.70					
Unspecified site viral/bacterial infection									
Yes	No	1.57	1.39	1.77					
**Asthmatic medication**									
ICS									
Yes	No	1.42	1.28	1.59					
LTRA									
Yes	No	1.08	0.99	1.18	0.87	0.79	0.95	0.002	− 1
DSCG AND SABA									
SABA with/without DSCG	No DSCG and SABA	1.65	1.48	1.83					
DSCG without SABA		1.22	0.82	1.81					

*ICS* inhaled corticosteroid, *DSCG* disodium cromoglycate, *LTRA* leukotriene receptor antagonist, *HR* hazard ratio, *LCL* 95% confidence lower-limit, *UCL* 95% confidence upper-limit.

*P-values are derived from Cox regression model with variables extracted by stepwise method.

### Risk score

**Figure 2:**
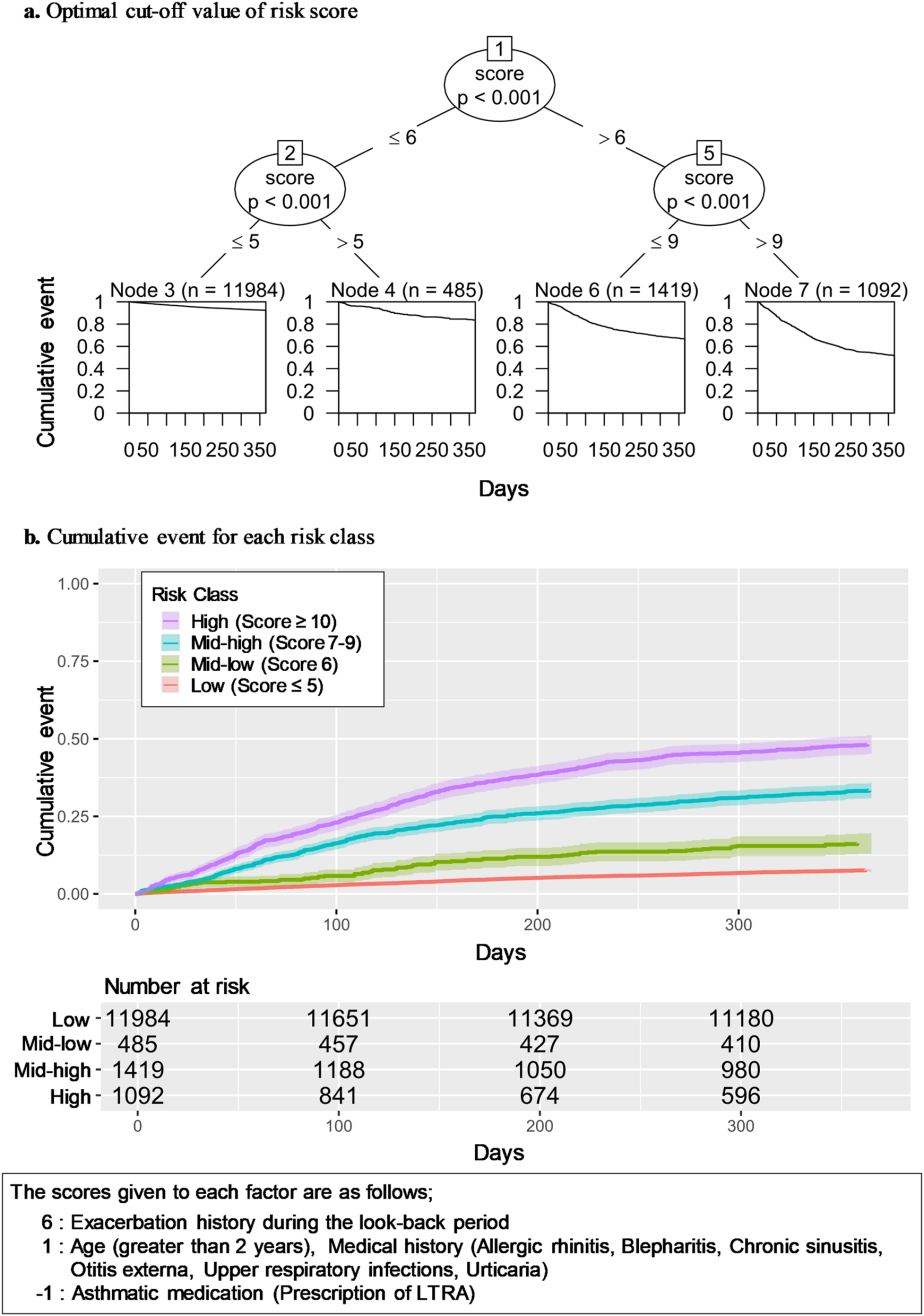
Optimal cut-off point of risk score and cumulative event among risk class. (**a**) Optimal cut-off point of risk score. (**b**) Difference in cumulative events between 4-level risk score.

The risk scores were calculated from the HRs of factors that were significantly associated with exacerbations in the multivariate Cox regression analysis (Table 3). The total risk score of each patient was then split by CART, resulting in cut-offs of 5, 6, and 9 points, with 5 points or less classified as low risk, 6 points as lower-middle risk, 7–9 points as higher-middle risk, and 10 points or more as high risk and the Harrell’s c-statistic for the model was 0.696 (Fig. 2a). The results of a tenfold cross-validation of the model for the risk score showed that the Accuracy and its 95% CI was 86.7% [85.5–87.9], and the AUC and its 95% CI was 0.700 [0.673–0.726]. The cumulative events for each level are shown by the Kaplan–Meier curve (Fig. 2b). Finally, the results of the sensitivity analysis using CART to classify the level of the variables showed that nine variables remained, with a c-statistic of 0.694 (Supplementary Fig. S1).

## Discussion

In this study, we extracted the factors significantly associated with the increase or decrease of asthma exacerbations in Japanese children with asthma by analyzing the claims data from health insurance. In addition, we scored the extracted factors and calculated a four-level risk classification (low to high risk) based on the total score according to the magnitude of risk, thereby assessing the combined increased risk.

The majority of the backgrounds, histories, and comorbidities that were extracted by multivariate Cox proportional hazards analysis and were significantly associated with exacerbations had already been reported in previous studies. Previous studies have shown that allergic rhinitis is associated with a high rate of asthma and impaired asthma control in pediatric patients with asthma^23,24^. Chronic sinusitis has also been reported as a risk factor^25^. With regard to the relationship between the upper and lower airways, it has been reported that antigen drops into the nasal cavity cause eosinophils to infiltrate the lower airways^26^, while in asthmatic patients without allergic rhinitis, the degree of eosinophil infiltration in the lower airways correlates with eosinophil infiltration of the nasal mucosa^27^. Allergic rhinitis and its impact on asthma (ARIA) have proposed the concept of “One airway, one disease”^28^, and the present study showed reasonable results.

While a number of factors can be interpreted in a way that is consistent with previous studies, some caution must be exerted in interpreting the factors that were associated with risk in this study. For otolaryngological disease, while previous studies have reported an association between otitis media and exacerbation risk, but not for otitis externa, in this study, on the contrary, outer ear disease remained a risk factor^29^. This may be due to confounding factors that co-occur with otitis externa; however, it was difficult to fully interpret the cause from the database.

As for treatment, the prescription of LTRA within 3 months prior to CED was associated with a lower risk of asthma exacerbation. In the PREVIA study, a randomized controlled trial evaluating the efficacy of LTRA in children evaluated montelukast or placebo for 1 year for virus-induced asthma exacerbations in children aged 2–5 years, and showed a 31.9% reduction in exacerbations in the montelukast group compared to the placebo group^30^. In this study, “with LTRA prescription” was defined as those prescribed for 28 days or more in the 3 months before CED. Additionally, the continuous use of LTRA may have been associated with a lower number of exacerbations, indicating that episodic viral wheezing may have been predominant in the eligible patients in this study. For DSCG and SABA, the frequent use of SABA prior to CED indicates a recent control status, which may suggest that the risk was higher than for children who did not use SABA.

The findings of the present study are consistent with those of previous studies in a variety of aspects. On the other hand, when considering the clinical role of “risk factors," the problem has been that asthma exacerbations are not caused by a single factor, but are expressed as a variety of phenotypes in combination with a multifactorial background, making prediction difficult. In the present study, we calculated the increased risk when multiple factors were combined using the following information: baseline age, exacerbations history in the past year, and the use of LTRA in the past 3 months; the risk of exacerbation of patients was classified into four levels using CART without laboratory or respiratory testing. Therefore, when this risk score algorithm is validated in real-world clinical practice, this method can be used as an alternative method for assessing the risk of exacerbation in children who are reluctant to undergo tests and their parents, and in situations where frequent examinations are difficult to perform due to insurance reimbursement. In addition, this approach may be useful for other diseases in the pediatric field and for studies of cancer and rare diseases that are difficult to conduct due to various challenges. A recent database study by Hatou et al. rated history of previous exacerbations as the highest risk score, similar to the present study. In that study, the third highest risk score was prescription of ICS/LABA; however, the study included 2–18-year-old, which differed in detail from our study in which the risk score was analyzed in Japan in patients under 5 years of age^31^. Furthermore, the results of the sensitivity analysis using CART showed that the c-statistic for the model was 0.694, suggesting that the performance and convenience of this simple method can be maintained.

The results and discussion of this study have several limitations. First, because the subjects of this study were limited to asthma patients belonging to a health insurance association and their family, the possibility that the regional distribution and patient background differ from those of the Japanese population cannot be denied. However, in this database, 20.2% of the subjects were under 15 years of age, which was not significantly different from the 12.4% of subjects younger than 15 years of age in Japan^32^. As for regional information, there were no major deviations in most prefectures at the prefecture level according to the Vital Statistics of the Ministry of Internal Affairs and Communications (Supplementary Table S2). Second, in the Japanese guidelines^33^, previous clinical studies have shown that the living environment, including pets, house dust, family smoking, regional differences (urban or rural) in high levels of dextran sulfate sodium, obesity, and pollen have a significant impact on asthma exacerbations. In addition, comorbidities such as GERD are listed as potential risks; however, these were not sufficiently extracted from this database. In this study, however, we were able to use only the data obtained from health insurance claims. Therefore, it is possible that information that was not obtained remains as unmeasured confounders. Third, there is a possibility that the diseases and outcomes specified in the claims records in this study do not reflect the actual diseases and outcomes. To ensure the validity of the outcomes, it is desirable to conduct an outcome validation. Finally, the c-statistic of the score-based model is limited to 0.696, which leaves room for improvement. It would be desirable to improve the model by combining actual clinical data.

## Conclusion

In this study, we extracted the factors associated with asthma exacerbations consistent with previous studies and evaluated the combined risk by using a retrospective cohort design using only Japanese health insurance claims data. The implication of this study is that combining each factor is more helpful in assessing the increased risk of asthma exacerbation than assessing each factor alone. In addition, the advantage of this method is that it does not require any special tests and can be easily applied to any medical institution. In the future, it will be desirable to validate these results in actual clinical practice and improve the accuracy of the method.

## Supplementary Information

Supplementary Figure S1.

Supplementary Table S1.

Supplementary Table S2.

## Data Availability

The data used in this study were provided by INTAGE Real World Inc. and the user agreement does not permit the data to be provided to third parties. Interested readers may contact the INTAGE Real World Inc. directly.

## Abbreviations

ATC: Anatomical therapeutic chemical classification system
AUC: Area under the receiver operating characteristic curve
CART: Classification and regression trees
DPC: Diagnosis procedure combination
DSCG: Disodium cromoglycate
HR: Hazard ratio
ICS: Inhaled corticosteroid
ICD-10: International Classification of Diseases, 10th Revision
LABA: Long-acting b2-adrenergic agonist
LTRA: Leukotriene receptor antagonist
OR: Odds ratio
SABA: Short acting beta-2 agonist
