# Supplementary figures and images for: Association of comorbidities and medications with risk of asthma exacerbation in pediatric patients: a retrospective study using Japanese claims data
Source: Sci Rep. 2022 Apr 1;12:5509. doi: 10.1038/s41598-022-08789-7 (PMC8975995; doi:10.1038/s41598-022-08789-7)

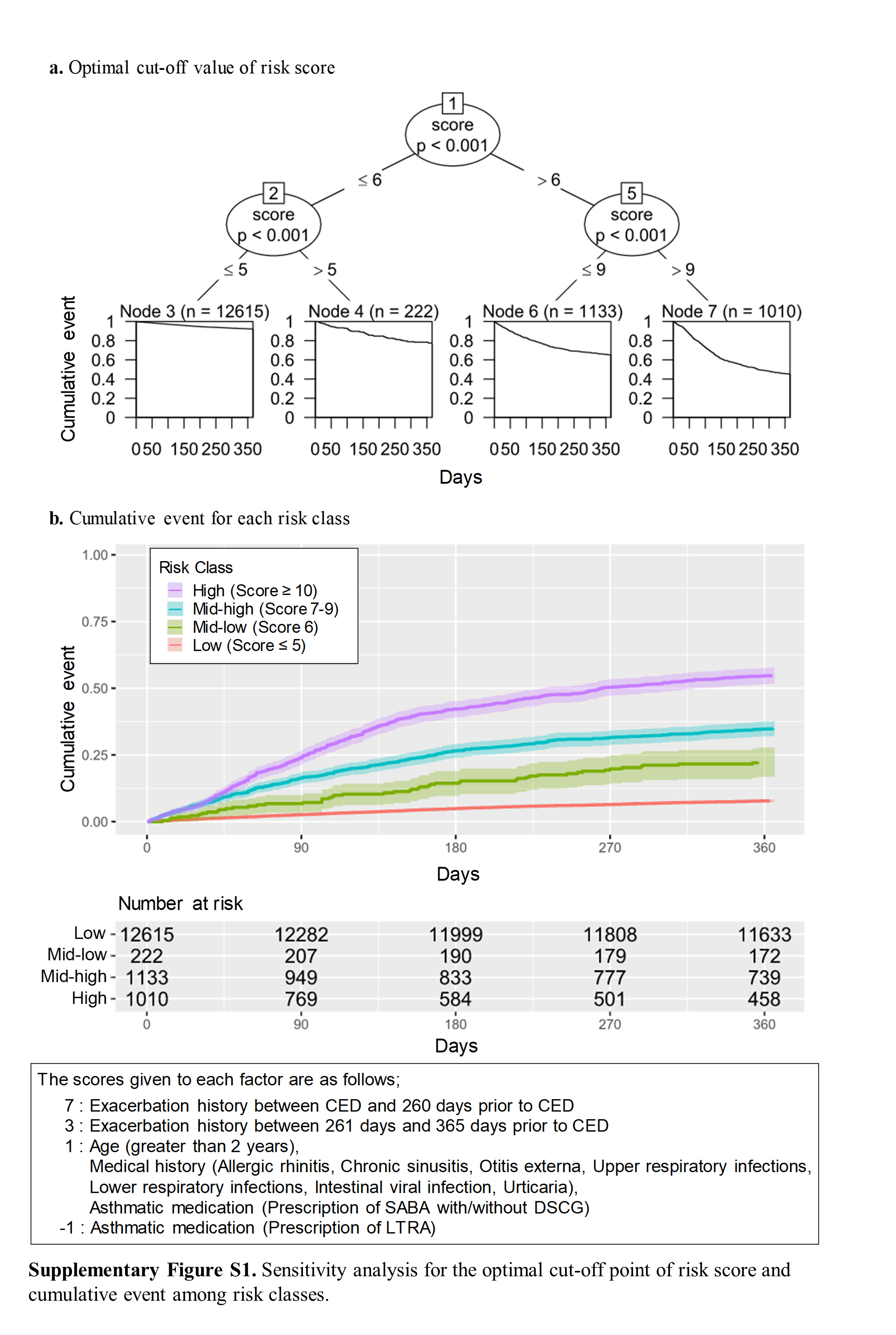

Supplement: Supplementary file 1 — Supplementary Figure S1. [file 41598_2022_8789_MOESM1_ESM.tif]
