# Supplementary material for: Association of comorbidities and medications with risk of asthma exacerbation in pediatric patients: a retrospective study using Japanese claims data
Source: Sci Rep. 2022 Apr 1;12:5509. doi: 10.1038/s41598-022-08789-7 (PMC8975995; doi:10.1038/s41598-022-08789-7)
Supplement: Supplementary file 2 — Supplementary Table S1. [file 41598_2022_8789_MOESM2_ESM.docx]

| Renamed term | ICD-10 code | Case (n) | |  | Control (n) | | OR | LCL | UCL |
| --- | --- | --- | --- | --- | --- | --- | --- | --- | --- |
|  |  | event | no-event |  | event | no-event |  |  |  |
| Allergic rhinitis | J30 Vasomotor and allergic rhinitis | 1,742 | 10,264 |  | 246 | 2,728 | 1.88 | 1.64 | 2.16 |
| Chronic sinusitis | J32 Chronic sinusitis | 772 | 3,803 |  | 1,216 | 9,189 | 1.53 | 1.39 | 1.69 |
| Otitis media/externa | H60 Otitis externa | 493 | 2,431 |  | 1,495 | 10,561 | 1.43 | 1.28 | 1.60 |
|  | H61 Other disorders of external ear | 847 | 4,519 |  | 1,141 | 8,473 | 1.39 | 1.26 | 1.53 |
|  | H65 Nonsuppurative otitis media | 446 | 2,052 |  | 1,542 | 10,940 | 1.54 | 1.37 | 1.73 |
|  | H66 Suppurative and unspecified otitis media | 939 | 5,552 |  | 1,049 | 7,440 | 1.20 | 1.09 | 1.32 |
| Blepharitis | B340 Adenovirus infection, unspecified site | 501 | 2,651 |  | 1,487 | 10,341 | 1.31 | 1.18 | 1.47 |
|  | H01 Other inflammation of eyelid | 246 | 1,313 |  | 1,742 | 11,679 | 1.26 | 1.09 | 1.45 |
| Upper respiratory infections | J00 Acute nasopharyngitis [common cold] | 932 | 5,356 |  | 1,056 | 7,636 | 1.26 | 1.14 | 1.38 |
|  | J01 Acute sinusitis | 679 | 3,552 |  | 1,309 | 9,440 | 1.38 | 1.25 | 1.52 |
|  | J02 Acute pharyngitis | 1,275 | 7,844 |  | 713 | 5,148 | 1.17 | 1.06 | 1.29 |
| Lower respiratory infections | J18 Pneumonia, organism unspecified | 777 | 3,419 |  | 1,211 | 9,573 | 1.80 | 1.63 | 1.98 |
|  | J205 Acute bronchitis due to respiratory syncytial virus | 50 | 174 |  | 1,938 | 12,818 | 1.90 | 1.38 | 2.61 |
|  | J208 Acute bronchitis due to other specified organisms | 26 | 74 |  | 1,962 | 12,918 | 2.31 | 1.48 | 3.63 |
|  | J209 Acute bronchitis, unspecified | 1,822 | 10,704 |  | 166 | 2,288 | 2.35 | 1.99 | 2.77 |
|  | J40 Bronchitis, not specified as acute or chronic | 593 | 3,525 |  | 1,395 | 9,467 | 1.14 | 1.03 | 1.27 |
| Influenza | J10 Influenza due to identified seasonal influenza virus | 432 | 2,498 |  | 1,556 | 10,494 | 1.17 | 1.04 | 1.31 |
|  | J11 Influenza, virus not identified | 982 | 5,889 |  | 1,006 | 7,103 | 1.18 | 1.07 | 1.29 |
| Dermatitis | L20 Atopic dermatitis | 763 | 4,395 |  | 1,225 | 8,597 | 1.22 | 1.11 | 1.34 |
|  | L30 Other dermatitis | 1,753 | 10,912 |  | 235 | 2,080 | 1.42 | 1.23 | 1.64 |
|  | L85 Other epidermal thickening | 1,310 | 8,078 |  | 678 | 4,914 | 1.18 | 1.06 | 1.30 |
| Urticaria | L50 Urticaria | 522 | 3,058 |  | 1,466 | 9,934 | 1.16 | 1.04 | 1.29 |
| Intestinal viral infection | A080 Rotaviral enteritis | 131 | 582 |  | 1,857 | 12,410 | 1.50 | 1.24 | 1.83 |
|  | A081 Acute gastroenteropathy due to Norwalk agent | 96 | 364 |  | 1,892 | 12,628 | 1.76 | 1.40 | 2.22 |
|  | A082 Adenoviral enteritis | 28 | 63 |  | 1,960 | 12,929 | 2.93 | 1.87 | 4.59 |
| Unspecified site viral/bacterial infection | B348 Other viral infections of unspecified site | 331 | 1,364 |  | 1,657 | 11,628 | 1.70 | 1.49 | 1.94 |
|  | B349 Viral infection, unspecified | 25 | 91 |  | 1,963 | 12,901 | 1.81 | 1.16 | 2.82 |

**Supplementary Table S1.** Aggregation criteria for original ICD-10 code to renamed term.

OR, odds ratio; LCL, 95% confidence lower-limit; UCL, 95% confidence upper-limit.
