# Supplementary material for: Association of comorbidities and medications with risk of asthma exacerbation in pediatric patients: a retrospective study using Japanese claims data
Source: Sci Rep. 2022 Apr 1;12:5509. doi: 10.1038/s41598-022-08789-7 (PMC8975995; doi:10.1038/s41598-022-08789-7)
Supplement: Supplementary file 3 — Supplementary Table S2. [file 41598_2022_8789_MOESM3_ESM.docx]

**Supplementary Table S2**. Differences in the population distribution between MediScope and population estimates by the Statistics Bureau.

| Prefecture | Study population | |  | Population estimates by the Statistics Bureau* | | Std-diff |  | Prefecture | Study population | |  | Population estimates by the Statistics Bureau* | | Std-diff |
| --- | --- | --- | --- | --- | --- | --- | --- | --- | --- | --- | --- | --- | --- | --- |
|  | n | % |  | x1000 | % |  |  |  | n | % |  | x1000 | % |  |
| Total | 14,980 |  |  | 4,963 |  |  |  |  |  |  |  |  |  |  |
| Hokkaido | 641 | 4.3% |  | 183 | 3.7% | 0.03 |  | Shiga | 355 | 2.4% |  | 63 | 1.3% | 0.08 |
| Aomori | 100 | 0.7% |  | 43 | 0.9% | 0.02 |  | Kyoto | 250 | 1.7% |  | 97 | 2.0% | 0.02 |
| Iwate | 111 | 0.7% |  | 44 | 0.9% | 0.02 |  | Osaka | 707 | 4.7% |  | 341 | 6.9% | 0.09 |
| Miyagi | 275 | 1.8% |  | 89 | 1.8% | 0.00 |  | Hyogo | 511 | 3.4% |  | 217 | 4.4% | 0.05 |
| Akita | 219 | 1.5% |  | 30 | 0.6% | 0.08 |  | Nara | 95 | 0.6% |  | 50 | 1.0% | 0.04 |
| Yamagata | 149 | 1.0% |  | 39 | 0.8% | 0.02 |  | Wakayama | 80 | 0.5% |  | 35 | 0.7% | 0.02 |
| Fukushima | 335 | 2.2% |  | 68 | 1.4% | 0.07 |  | Tottori | 48 | 0.3% |  | 23 | 0.5% | 0.02 |
| Ibaraki | 281 | 1.9% |  | 109 | 2.2% | 0.02 |  | Shimane | 38 | 0.3% |  | 27 | 0.5% | 0.05 |
| Tochigi | 254 | 1.7% |  | 76 | 1.5% | 0.01 |  | Okayama | 125 | 0.8% |  | 77 | 1.6% | 0.07 |
| Gunma | 363 | 2.4% |  | 72 | 1.5% | 0.07 |  | Hiroshima | 213 | 1.4% |  | 118 | 2.4% | 0.07 |
| Saitama | 1,096 | 7.3% |  | 284 | 5.7% | 0.06 |  | Yamaguchi | 165 | 1.1% |  | 51 | 1.0% | 0.01 |
| Chiba | 904 | 6.0% |  | 236 | 4.8% | 0.06 |  | Tokushima | 63 | 0.4% |  | 27 | 0.5% | 0.02 |
| Tokyo | 1,749 | 11.7% |  | 534 | 10.8% | 0.03 |  | Kagawa | 68 | 0.5% |  | 38 | 0.8% | 0.04 |
| Kanagawa | 1,490 | 10.0% |  | 362 | 7.3% | 0.09 |  | Ehime | 122 | 0.8% |  | 51 | 1.0% | 0.02 |
| Niigata | 452 | 3.0% |  | 82 | 1.7% | 0.09 |  | Kochi | 83 | 0.6% |  | 25 | 0.5% | 0.01 |
| Toyama | 53 | 0.4% |  | 38 | 0.8% | 0.06 |  | Fukuoka | 626 | 4.2% |  | 221 | 4.5% | 0.01 |
| Ishikawa | 68 | 0.5% |  | 45 | 0.9% | 0.06 |  | Saga | 133 | 0.9% |  | 35 | 0.7% | 0.02 |
| Fukui | 35 | 0.2% |  | 31 | 0.6% | 0.06 |  | Nagasaki | 231 | 1.5% |  | 55 | 1.1% | 0.04 |
| Yamanashi | 111 | 0.7% |  | 30 | 0.6% | 0.02 |  | Kumamoto | 215 | 1.4% |  | 76 | 1.5% | 0.01 |
| Nagano | 128 | 0.9% |  | 79 | 1.6% | 0.07 |  | Oita | 140 | 0.9% |  | 45 | 0.9% | 0.00 |
| Gifu | 128 | 0.9% |  | 79 | 1.6% | 0.07 |  | Miyazaki | 205 | 1.4% |  | 46 | 0.9% | 0.04 |
| Shizuoka | 312 | 2.1% |  | 145 | 2.9% | 0.05 |  | Kagoshima | 279 | 1.9% |  | 70 | 1.4% | 0.04 |
| Aichi | 454 | 3.0% |  | 327 | 6.6% | 0.17 |  | Okinawa | 386 | 2.6% |  | 83 | 1.7% | 0.06 |
| Mie | 134 | 0.9% |  | 70 | 1.4% | 0.05 |  |  |  |  |  |  |  |  |

*Aged 0 to 4 years old,
†Standardized difference
